# Editorial of the Special Issue “Molecular Research and Recent Advances in Diabetic Retinopathy”

**DOI:** 10.3390/biomedicines12081879

**Published:** 2024-08-16

**Authors:** Tomislav Bulum, Martina Tomić

**Affiliations:** 1 Department of Diabetes and Endocrinology, Vuk Vrhovac University Clinic for Diabetes, Endocrinology and Metabolic Diseases, Merkur University Hospital, 10000 Zagreb, Croatia; 2 School of Medicine, University of Zagreb, Šalata 3, 10000 Zagreb, Croatia; 3 Department of Ophthalmology, Vuk Vrhovac University Clinic for Diabetes, Endocrinology and Metabolic Diseases, Merkur University Hospital, Dugi dol 4a, 10000 Zagreb, Croatia

Despite increasing awareness of diabetes and its devastating complications, it remains the most rapidly escalating global health issue [1]. It was estimated to have affected 537 million people and accounted for 12.2% of all global deaths in 2021, with projections indicating that this number will rise to 783 million by 2045 [2]. The cost of diabetes treatment and its complications has also seen a dramatic rise, with global health expenditure reaching USD 966 billion in 2021 [2].

Diabetic retinopathy (DR), a microvascular complication of diabetes, remains the leading cause of blindness among the adult working population [3]. Studies indicate that about 22% of patients with diabetes will develop DR, with about 10% experiencing vision-threatening forms such as proliferative DR (PDR) and diabetic macular edema (DME) [4]. Early diagnosis of DR is crucial in preventing these severe forms of the condition. The World Health Organization’s global action plan highlights the urgent need to reduce the prevalence of preventable blindness. Recent advancements in treatment methods, including anti-vascular endothelial growth factor (anti-VEGF) agents and steroids, have contributed to a decline in diabetes-related blindness over the past few years [5,6]. Nevertheless, effective control of systemic risk factors and regular screening remain the primary strategies for preventing the development and progression of DR [7,8].

Sodium-glucose cotransporter 2 (SGLT2) inhibitors and glucagon-like-peptide-1 receptor (GLP-1) agonists represent new antidiabetic drugs that are now considered fundamental in the treatment of type 2 diabetes mellitus because of their favorable cardioprotective effects, including a decreased risk of diabetic nephropathy progression [9]. Although these medications have a favorable effect on diabetic nephropathy as a microvascular disease, they have neutral or even negative effects on DR, another diabetic microvascular disease. Patients treated with GLP-1 agonists have a higher rate of progression of PDR and a risk of new-onset DME, so these patients must be closely monitored for retinopathy status to decrease their risk of vision-threatening complications [10,11].

Considering all these factors, additional efforts are needed to reduce the risk of blindness and disability in patients with diabetes, and this Special Issue aims to present the latest molecular research and recent advances in DR and DME, comprising eight research articles and six review articles.

The first research article found associations between the severity of DR and the DNA methylation levels of the genes in patients with type 1 diabetes, suggesting a potential influence of disease progression and associated factors on the methylation status of investigated genes.

The second research article reports a retrospective cohort study that included a large cohort of subjects and investigated the relationship between DR and age-related macular degeneration, a primary cause of vision impairment in older adults. The results of the study suggest a multifaceted relationship between DR and age-related macular degeneration, regardless of comorbid conditions, and heightened awareness of the increased risk of age-related macular degeneration in patients with DR is needed.

The third research article investigated stem cell therapy in the treatment of DR. Sirolimus-pretreated mesenchymal stem cells were subconjunctivally injected in diabetic rats with DR. The treatment resulted in an increase in electroretinogram amplitude with a reduction in the retinal thickness at a histological level. Sirolimus-pretreated mesenchymal stem cells can potentially be used in DR treatment, and the subconjunctival route of administration can be effective for the treatment of DR.

The fourth research article explores the fastest-growing field in the diagnostics of DR. It is well known that screening every patient with diabetes for DR with standard ophthalmological examinations is not possible, and not all patients with diabetes must be checked by an ophthalmologist. That is not possible and represents an inefficient use of resources. Currently, several low-cost fundus cameras handled by educated nurses or trained photographers are widely available, and fundus photography telemedicine reviewed by ophthalmologists or artificial intelligence (AI)-based automated system has become a promising option for DR screening and diagnosis [12,13,14,15,16]. In this study, color fundus photography with a TANG hand-held fundus camera was conducted by the ophthalmology nurse and graded using AI-based automated software and an independent ophthalmologist. The results suggest that AI-based systems are more sensitive than human graders and could be safe to use in clinical practice to screen for DR. Moreover, the percentage of subjects with advanced stages of DR (severe NPDR and PDR) who were detected using the hand-held fundus camera and AI-based grading system corresponded with absolute accuracy to those detected by the clinical examination and standard camera. In support of these results, the fifth research article reports a pilot DR screening program in Oslo. The researchers obtained color fundus photographs and found that over 50% of patients had some degree of DR, and 62.8% had never had an eye exam. The results indicate an urgent need to implement a systematic DR screening program in the Oslo region.

The sixth research article found associations between the Solute Carrier Family 22 Member 3 (SLC22A3) gene polymorphism and DR in the Slovenian population of subjects with type 2 diabetes mellitus. Studies with a larger sample are necessary to confirm this interaction.

The seventh research article focused on inflammation caused by hyperglycemia and its effects on DR. The researchers used neutrophil traps, which have an inflammatory mechanism of neutrophils associated with inflammation in chronic diseases, and found that neutrophil traps are associated with glucoregulation, renal function, and the development of DR.

Finally, the eighth research article confirmed the well-known relationship between DR and diabetic nephropathy, which are the two most important microvascular complications of diabetes. They thoroughly investigated that connection and additionally found that diabetic nephropathy severity may be associated with the development of DME.

The first review thoroughly investigates developments in multimodal imaging and functional tests for early DR detection such as retinal microvascular tortuosity, caliber, fractal dimension, optical coherence tomography with and without angiography, multifocal electroretinography, multifocal pupillographic objective perimetry, visual-evoked potential, microperimetry, contrast sensitivity, artificial intelligence techniques, and hematological parameters such as the neutrophil–lymphocyte ratio and neutrophil extracellular traps.

The second review focuses on several types of natural compounds, such as alkaloids, phenols, terpenoids, flavonoids, saponins, vitamins, and saccharides, in the prevention of DR, because those natural compounds have antioxidant, anti-neovascular, antiapoptotic, and anti-inflammatory effects. However, the lack of a wider use of natural products in clinical practice and the potential risk of toxicity call for prospective clinical studies to evaluate the therapeutic effects of natural products in the development and progression of DR.

The third comprehensive narrative review details the therapeutic potential of aldose reductase inhibitors in treating DR. It is well known that aldose reductase, the enzyme involved in the polyol pathway and aggravated by hyperglycemia, plays an important role in the pathogenesis of chronic complications of diabetes, as well as DR. Since most studies that investigated aldose reductase inhibitors were withdrawn due to unfavorable side effects, efforts must be taken to identified novel aldose reductase inhibitors drugs or to use natural compounds with proven aldose reductase inhibitor effects.

The fourth review focused on the impact of modern antidiabetic treatment on endothelial progenitor cells, which are cells that have beneficial effects on endothelium and can restore vascular function. Treatment with traditional antidiabetic drugs, such as insulin, metformin, pioglitazone, sulphonylureas, and dipeptidyl peptidase-4 inhibitors, have beneficial effects on endothelial progenitor cells, while there is limited evidence for the new cardio-reno-protective drugs SGLT2 inhibitors and GLP-1 agonists.

The fifth review summarized advances and perspectives about the molecular basis of DR, primarily focusing on advanced glycation end-products, asymmetric dimethylarginine, vascular endothelial growth factor (VEGF), epigenetic regulation mediated by microRNAs, and endothelin-1.

Finally, the sixth review focuses on the role of inflammation in the pathogenesis of DME and age-related macular degeneration and emphasizes the anti-inflammatory effects of dazdotuftide, which might be, along with anti-VEGF therapy, a good treatment option for the management of these conditions.

In conclusion, this Special Issue provides a comprehensive overview of recent developments and molecular research on DR as well as DME and aims to improve our understanding and treatment of these harmful complications of diabetes. This new knowledge will facilitate the diagnosis of these conditions and may be the basis for future therapeutic approaches.
